# Supplementary material for: Content-rich biological network constructed by mining PubMed abstracts
Source: BMC Bioinformatics. 2004 Oct 8;5:147. doi: 10.1186/1471-2105-5-147 (PMC528731; doi:10.1186/1471-2105-5-147)
Supplement: Additional File 2 — The original results of the above study (non-essential files are deleted to keep the file size under the limit set by BMC bioinformatics). [file 1471-2105-5-147-S2.bz2 › chilibotAdditionalFile2/dip10/564ID10574949E10925/html/FGF8_FGFR3.html]

 


 **FGF8** and **FGFR3** 
  
Found 13 abstracts in PubMed, retrieved 10.  
 

 What does Google say? 
 PDF only 
| .edu only 

---

**Interactive relationship** (e.g. stimulation, inhibition, etc)

**Neutral relationship**- In contrast,  **FGFR3**  bound well to  **FGF8**  and FGF9 but poorly to FGF2.  Ref: 10574949 J Biol Chem, 1999
- First, we demonstrated that FGF receptor 1 Fgfr1 and  **Fgfr3** , expressed differentially in the developing brain, possess an equivalent potential to induce the regionally distinct  **Fgf8**  responsive genes, depending on the anterior posterior dimension of the brain.  Ref: 11782403 Development, 2002

**Non-interactive relationship** (e.g. studied together, co-existance, homology, etc.)

- Although two of the receptors which  **FGF8**  binds to FGFR2 IIIc,  **FGFR3**  IIIc are not expressed in breast cancer cells, an autocrine activation loop is possible since expression of fibroblast growth factor receptor FGFR 4 and FGFR1 are retained in malignant epithelial cells.  Ref: 10023681 Oncogene, 1999
- Mutations in the genes for fibroblast growth factor FGF 8  [ **FGF8** ]  and receptors FGFR1, FGFR2, and  **FGFR3**  were not detected.  Ref: 10664233 Eur J Pediatr, 2000
- Further, analysis of  **fgfr3**  expression in mutants deficient in the formation of the midbrain hindbrain boundary MHB, noi and ace, demonstrated that in the absence of Pax2.1 and  **FGF8**  activity, the expression domains of  **FGFR3**  expand into the MHB, tegmentum, cerebellum and optic tectum, which are the affected structures in these mutants.  Ref: 11287195 Mech Dev, 2001
